# Implications of a peroxisome proliferator-activated receptor alpha (PPARα) ligand clofibrate in breast cancer

**DOI:** 10.18632/oncotarget.6402

**Published:** 2015-11-26

**Authors:** Karthic Chandran, Sudeshna Goswami, Neelam Sharma-Walia

**Affiliations:** ^1^ H. M. Bligh Cancer Research Laboratories, Department of Microbiology and Immunology, Chicago Medical School, Rosalind Franklin University of Medicine and Science, North Chicago, Illinois, U.S.A

**Keywords:** breast cancer, fatty acid synthase, COX-2, PPAR, prostaglandin E2

## Abstract

Inflammatory and invasive breast cancers are aggressive and require better understanding for the development of new treatments and more accurate prognosis. Here, we detected high expression of PPARα in human primary inflammatory (SUM149PT) and highly invasive (SUM1315MO2) breast cancer cells, and tissue sections of human breast cancer. PPAR*α* ligands are clinically used to treat dyslipidemia. Among lipid lowering drugs clofibrate, fenofibrate and WY14643, clofibrate showed high chemo-sensitivity towards breast cancer cells. Clofibrate treatment significantly induced PPARα DNA binding activity, and remarkably reduced cyclooxygenase-2/PGE2 and 5-lipoxygenase/LTB4 inflammatory pathways. Clofibrate treatment reduced the proliferation of breast cancer cells probably by inhibiting NF-κB and ERK1/2 activation, reducing cyclinD1, cyclinA, cyclinE, and inducing pro-apoptotic P21 levels. Surprisingly, the expression of lipogenic pathway genes including SREBP-1c (sterol regulatory element-binding protein-1c), HMG-CoA synthase, SPTLC1 (serine palmitoyltransferase long-chain), and Acyl-CoA oxidase (ACO) decreased with a concurrent increase in fatty acid oxidation genes such as CPT-1a (carnitine palmitoyltransferase 1a) and SREBP-2 (Sterol regulatory element-binding protein-2). Clofibrate treatment induced secretion of free fatty acids and effectively decreased the level of phosphorylated active form of fatty acid synthase (FASN), an enzyme catalyzing de novo synthesis of fatty acids. High level of coactivators steroid receptor coactivator-1 (SRC-1) and histone acetylase CBP-300 (CREB binding protein-300) were observed in the nuclear complexes of clofibrate treated breast cancer cells. These findings implicate that stimulating PPARα by safe, well-tolerated, and clinically approved clofibrate may provide a safer and more effective strategy to target the signaling, lipogenic, and inflammatory pathways in aggressive forms of breast cancer.

## INTRODUCTION

Breast cancer is the most common cancer and the second leading cause of death from malignancy in women in the United States. Highly metastatic inflammatory breast cancer (IBC) is a rare and lethal form of breast cancer affecting roughly 1–6% of all breast cancer patients [1]. IBC is treated using a multimodal approach but patients have a poor prognosis, and have a high mortality rate, due to the ineffective and toxic chemotherapy [1]. Thus, there remains an urgent need of safe and efficacious drugs that can combat this aggressive breast cancer.

The peroxisome proliferator-activated receptors (PPARs) are ligand-activated transcription factors belonging to the superfamily of nuclear hormone receptors. PPARs act as key transcriptional regulators of lipolytic pathways such as mitochondrial, peroxisomal, and microsomal fatty acid oxidation (FAO), and play an important role in nutrient homeostasis, and lipid metabolism [2, 3]. PPARs perform their activity via formation of heterodimers with the nuclear receptor, RXR (Retinoid X receptor), followed by binding to specific DNA-response elements in target genes known as peroxisome proliferator response elements (PPREs) [2, 3]. Three PPAR subtypes, PPAR*α*, PPAR*β/δ*, and PPAR*γ,* are dynamically regulated at multiple molecular levels. Since its discovery in the early 1990s, PPAR*α* has emerged as a crucial transcriptional regulator of numerous metabolic and inflammatory processes [2, 3]. PPAR*α* is the master regulator of hepatic lipid metabolism, lipoprotein metabolism, and also known to activate growth factor signaling pathways, liver inflammation, energy homeostasis, cholesterol and bile acids, xenobiotics, and amino acid metabolism [2, 3]. Transcriptional activity of PPARs is controlled by both the availability of PPAR ligands and by interactions with protein coactivators and corepressors also known as “coregulators” that are recruited into transcriptional complexes and subsequently activate/suppress gene expression [4]. Because coactivators such as steroid receptor coactivator-1 (SRC-1), p300 kDa/CREB binding protein (p300/CBP) affect chromatin configuration and recruit protein complexes to serve as a link between the PPAR and the transcriptional apparatus, they are critical fine-tuning proteins for many aspects of classic PPAR transcriptional function and when coregulator expression goes wrong, pathogenesis can occur. Targeting coregulator function could be considered as a treatment strategy in conjunction with or independently of selective PPAR modulation. One of the major challenges lying ahead is to gain a better understanding of the molecular mechanism underlying the downregulation of gene expression by PPAR*α*. There is need to improve insight into the specific mechanisms and pathways of endogenous PPAR*α* activation in order to better link the functional consequences of PPAR*α* activation to induction of PPAR*α* responsive target genes.

PPARs are involved in various cellular functions including proliferation, metabolic regulation, and thus making PPAR agonists promising drugs for the treatment of lung cancer, endometrial cancer, and ovarian cancer [2, 3]. Pharmacological synthetic agonists (ligands) of PPAR*α* such as plasticizers, herbicides, and fibrates, including gemfibrozil, bezafibrate, clofibrate, fenofibrate, and WY14643 are clinically used in the treatment of dyslipidemia, and their safety, tolerance, and minimal side effects being well documented [2, 3]. PPAR-α is a pleiotropic regulator best known as a transcriptional regulator of lipid and glucose metabolism but has also accumulated its importance in diverse functions such as keratinocyte differentiation, wound healing [5] and in skin diseases including benign epidermal tumors, melanoma tumors, papillomas, acne vulgaris and psoriasis [6–10]. PPAR-α ligands have been reported to have anti-metastatic activity *in vivo* against skin cancer in experimental models [9]. PPAR*α* is considered a crucial fatty acids sensor, and natural ligands of PPAR*α* include a variety of fatty acids such as linoleic acid, arachidonic acid (AA), acyl-CoAs, oxidized fatty acids, eicosanoids, endocannabinoids, prostaglandin J2 (PGJ2), phytanic acid, and leukotriene B4 (LTB4) [2, 3, 11]. PPAR-α activation increases the expression of a wide range of enzymes that promote fatty acid and triglyceride oxidation including acyl-CoA oxidase (ACO), CPT1, malonyl-CoA decarboxylase (MLYCD), and downregulates FASN activity, and SREBP-1c involved in *de novo* fatty acid synthesis [2, 3, 12, 13]. Since PPAR*α* activation is considered to be valuable for the prevention and improvement of metabolic syndrome, we hypothesized that PPAR*α* activation plays a protective role in debilitating inflammatory and invasive breast cancer progression. Here, we chose to focus on two triple-negative breast cancer (TNBC) cell lines SUM149PT and SUM1315MO2. The SUM149PT cell line was developed from Invasive Ductal Carcinoma from a patient with inflammatory breast cancer. This cell line is immortal and expresses luminal cytokeratins 8, 18, and 19 consistent with their origin from luminal breast epithelial cells. SUM149PT has been known to form tumors in nude mice [14]. The SUM1315MO2 cell line was developed from a highly invasive breast cancer specimen of patient with skin metastasis of infiltration ductal carcinoma that was grown for two transplant generations in immune-deficient mice before being explanted into culture [14]. SUM149PT and SUM1315MO2 cell lines are BRCA1 (breast cancer 1, early onset) mutated [15]. BRCA1 is normally expressed in the cells of breast and other tissue, where it helps repair damaged chromosomal DNA damage or destroy cells if DNA cannot be repaired [15]. In this study, we investigated the anti-tumorigenic, anti-lipogenic, and anti-inflammatory potential of PPAR*α* agonist clofibrate in SUM149PT and SUM1315MO2 triple-negative breast cancer cell lines.

## RESULTS

### Breast cancer cells express higher levels of PPARα as compared to HMEC cells

**Figure 1:**
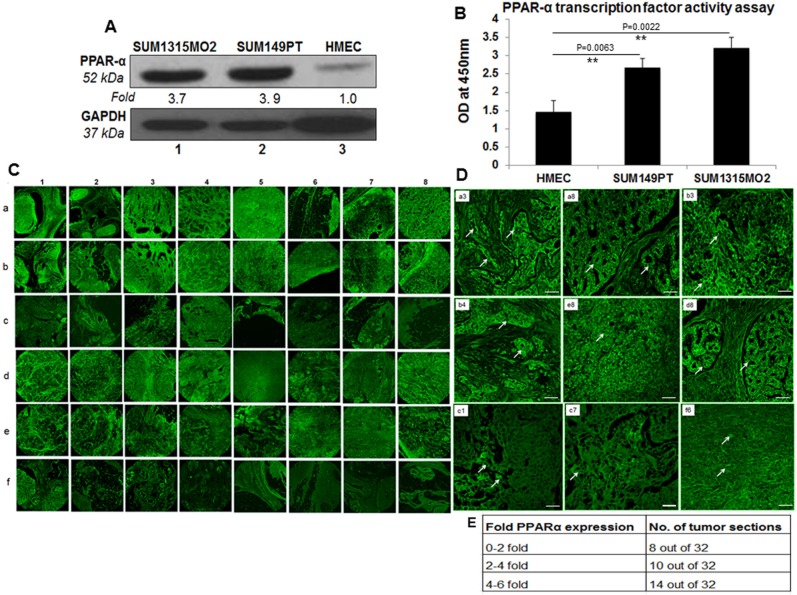
PPARα levels in human breast cancer cell lines and tissue samples **A.** Lysates prepared from SUM1315MO2, SUM149PT, and HMEC cells were tested for the protein levels of PPARα. The blots were re-probed with anti-GAPDH antibody to confirm equal loading. A representative blot from three independent experiments is shown. **B.** PPRE binding activity of PPARα. HMEC, SUM149PT and SUM1315MO2 nuclear extracts were prepared and tested for their ability to bind specifically to the immobilized PPRE in an ELISA based test. Results represent the absorbance measured at 450nm, and are the mean ± SD of three separate experiments from three different preparations for each condition. ** represent statistically highly significant. **C.** 16 breast cancer tissue samples, in duplicates (a,b,d,e) along with their controls (c,f) were analyzed by IHC staining for PPARα. Magnification for the panels is 4X. **D.** Magnified view (60X) of PPARα staining in selected human breast cancer tissue samples. White arrows indicate PPARα staining. Scale bar = 20 μm. **E.** Fold change of PPARα staining in tumor sections. Sections shown in C were further analyzed by measuring fold expression using densitometric analysis. Fold expression was calculated by considering staining in control sections as one fold.

Compared to HMEC cells, increased expression of PPARα was observed in SUM149PT (3.9-fold) and SUM1315MO2 (3.7-fold) breast cancer cells (Figure 1A). Similarly, the nuclear extracts prepared from breast cancer cells showed significantly increased transcriptional activity of PPARα binding to PPARα response element (PPRE) than the HMEC nuclear extracts (Figure 1B).

### PPARα expression is significantly elevated in the breast cancer tissue

To extend our previous *in vitro* observations, we analyzed the breast tissue sections of healthy subjects and breast cancer patients for the presence of PPARα by immunofluorescence staining using anti-PPARα antibody. Abundant PPARα expression was detected in breast cancer tissue sections (Figures 1C, panels a, b and d, e) compared to the normal healthy control tissue sections (Figure 1C, panel c and f). There was a consistent high expression of PPARα in the breast cancer tissue samples (Figures 1C, panels a1, b1, d1, e1, e2, a3, b3, d3, a4, b4, d4, a5, a7, a8, b8, d8, and e8) when compared to the normal healthy control tissue samples counterparts in panels c and f. However, there were exceptions where the healthy control tissue samples showed abundant expression of PPARα (Figure 1C, panel f1, f8). We next evaluated the fold change in PPARα expression in all 32 sections by densitometry analysis using ImageJ software. A 0–2, 2–4, 4–6 fold induction in PPARα expression was observed in 8, 10 and 14 tumor sections, respectively (Figure 1E). Abundant PPARα expression in breast cancer tissue as compared to healthy control tissue is clearly evident in the detailed magnified images provided in Figure 1D. Collectively these results highlight the presence of PPARα expression in human breast cancer tissues.

### PPARα expression is significantly elevated in the tissue sections of inflammatory breast cancer (IBC) patient tissue sections

**Figure 2:**
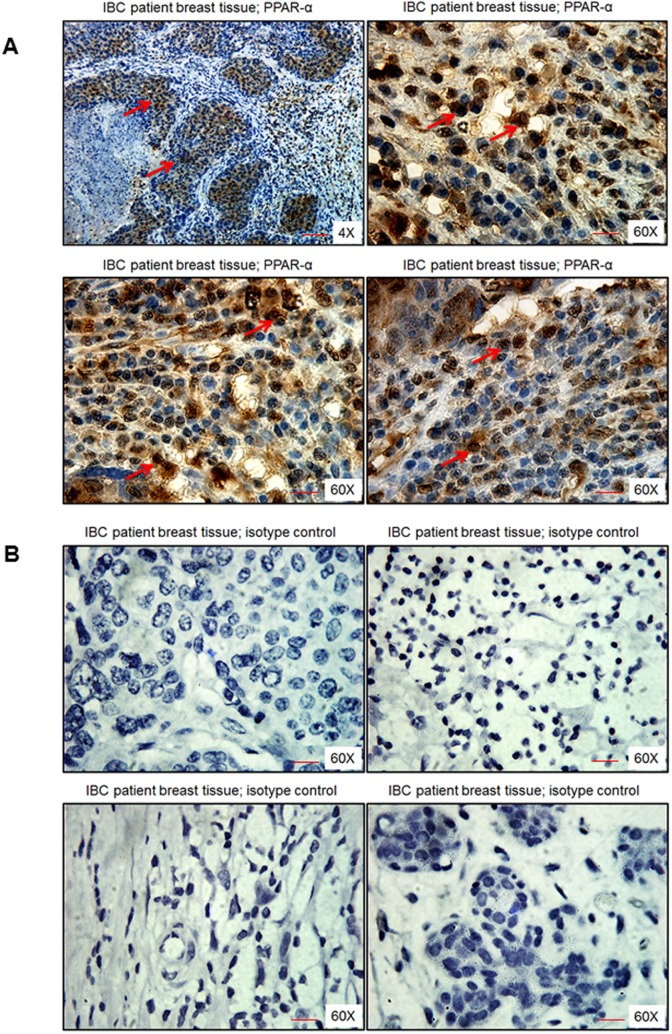
PPARα levels in human breast tissue obtained from IBC patients Breast tissue section was stained with either **A.** PPARα antibody or **B.** isotype control antibody for PPARα. Magnification 4X and 60X. Red arrows indicate PPARα staining. Scale bar = 20 μm.

IBC patient breast tissue sections showed dense brown nuclear staining of PPARα, especially in the ductal regions of cancer specimens as observed in Figure 2A. Specificity of PPARα staining was confirmed by using an isotype control antibody for PPARα, which did not show any brown staining (Figure 2B).

### 20 μM clofibrate was appropriate to treat SUM149PT and SUM1315MO2 cells

**Figure 3:**
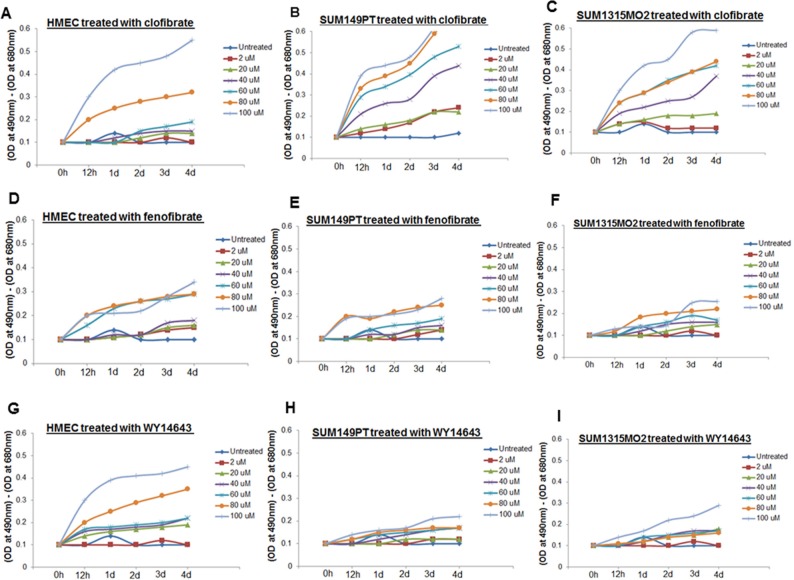
Effect of clofibrate, fenofibrate, and WY14643 treatment on cytotoxicity HMEC, SUM149PT, and SUM1315MO2 cells were untreated or treated with **A–C.** clofibrate, **D–F.** fenofibrate, and **G–I.** WY14643 for differing time points at various concentrations as indicated. Supernatants were collected from untreated and drug treated cells to measure the level of LDH release by spectrophotometer at 490 and 680 nm.

Clofibrate and fenofibrate have been shown to activate PPAR*α* with 10-fold selectivity over PPAR*γ* [16]. WY14643, the 2-aryl-thioacetic acid analogue of clofibrate is a potent PPAR*α* agonist as well as a weak PPAR*γ* agonist. Clofibrate, fenofibrate, and WY14643 activate PPARs but the direct binding of these drugs with PPARs has not been demonstrated [16]. Three PPARα agonist activators including clofibrate, fenofibrate, and WY14643 were used for treatment to evaluate their cytotoxicity in SUM149PT, SUM1315MO2, and HMEC cells. As the results show fenofibrate was minimally cytotoxic to any of the breast cancer cells but was toxic to control HMEC cells (Figure 3D–3F). WY14643 treatment was highly cytotoxic to healthy control HMEC cells (Figure 3G) but had no effect on the viability of breast cancer cells even at higher doses tested (Figure 3H and 3I). In contrast, clofibrate was significantly cytotoxic at high concentrations (60–100 μM) but provided optimal growth conditions up to 20 μM for all the cell types tested (Figure 3A–3C).

**Figure 4:**
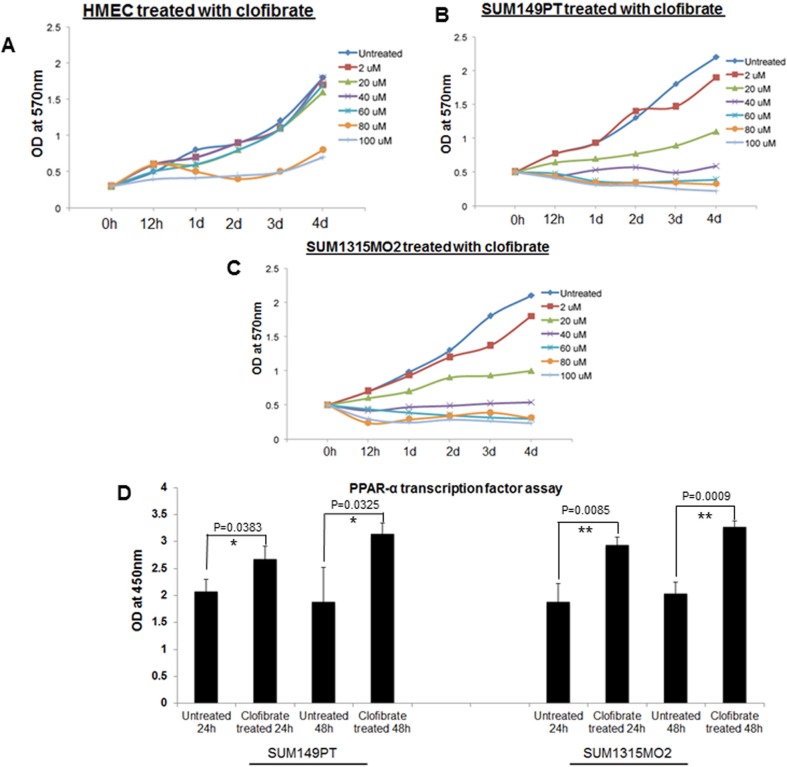
Effect of clofibrate treatment on cell proliferation MTT cell proliferation assay was performed in **A.** HMEC, **B.** SUM149PT, and **C.** SUM1315MO2 cells, which were treated with clofibrate as indicated, and the levels of tetrazolium MTT was measured by a spectrophotometer at 570nm. **D.** Effect of clofibrate treatment on PPRE binding activity of PPARα in breast cancer cell lines. SUM149PT and SUM1315MO2 cells were treated for 24 h and 48 h with 20 μM clofibrate and nuclear extracts were prepared and tested for PPRE binding activity. Results represent the absorbance detected at 450nm. Readings are the mean ± SD of three separate experiments from three different preparations for each condition. * denotes statistically significant and ** represents statistically highly significant.

Once clofibrate was selected as the PPARα ligand of choice, it was important to fine-tune the proper concentration and duration of the treatment that would be optimal to study cell proliferation kinetics and the cell cycle in various cell types. SUM149PT, SUM1315MO2, and HMEC cells were treated with various concentrations of clofibrate for different time points as indicated and an MTT assay was performed. The MTT assay was used to measure the differences in the mitochondrial activity of viable and growth arrested normal and breast cancer cells. In MTT assays, we observed significant growth arrest of SUM149PT and SUM1315MO2 cells at concentrations higher than 40 μM (Figure 4B and 4C). MTT assay results (Figure 4A–4C) supported results obtained from cytotoxicity assays (Figure 3), and we concluded that 20 μM clofibrate is an ideal PPARα ligand concentration for up to 48 hours treatment (Figure 4A). We tested if 20 μM clofibrate would actually augment binding to PPRE in the promoter region and activate the target genes through the DNA binding domain with a PPARα. To evaluate PPRE binding, we used a PPARα transcription factor assay. SUM149PT and SUM1315MO2 cells showed statistically significant increased PPARα transcriptional activity upon treatment with 20 μM of clofibrate at 24 h and 48 h (Figure 4D).

### Clofibrate treatment reduces COX-2 pathway enzymes in SUM149PT and SUM1315MO2 cells

**Figure 5:**
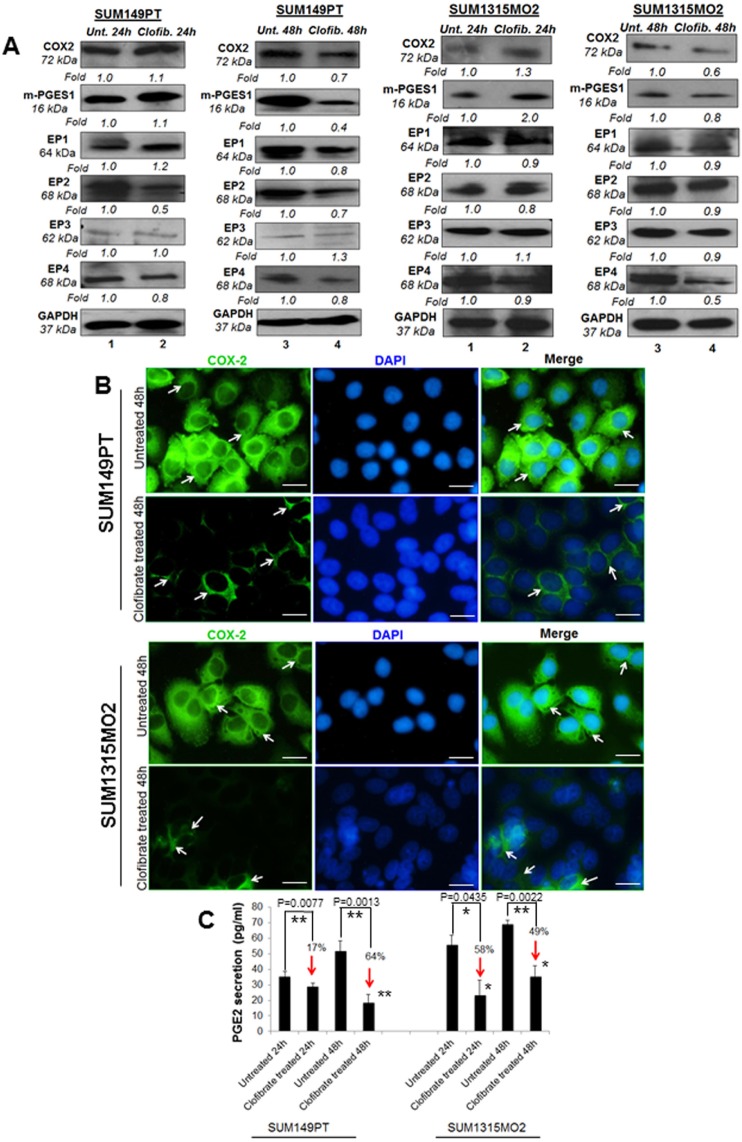
Effect of clofibrate treatment on the cyclooxygenase pathway Lysates prepared from **A.** SUM149PT and SUM1315MO2 cells untreated or treated with 20 μM clofibrate for 24 h and 48 h were Western blotted for COX-2, mPGES-1, EP1, EP2, EP3, EP4 and then stripped and re-probed with anti-GAPDH antibody to confirm equal loading. **B.** COX-2 immunostaining in SUM149PT and SUM1315MO2. SUM149PT and SUM1315MO2 cells were untreated or treated with 20 μM clofibrate for 48 h in eight-well chamber slides and then collected, permeabilized, and stained with an anti-COX-2 monoclonal antibody. Magnification, 40X. DAPI (Blue) was used as a nuclear stain and merged with COX-2 staining. Scale bar = 20 μm. **C.** Effect of clofibrate treatment on PGE2 secretion. Cell free culture supernatants of SUM149PT and SUM1315MO2 untreated or treated with 20 μM clofibrate for 24 h and 48 h were used to measure PGE2. Percent inhibition of PGE2 secretion was calculated by considering the secretion from untreated cells as 100%. * denotes statistically significant and ** represents statistically highly significant.

Since we observed higher levels of PPARα in breast cancer cells versus HMEC cells, we next evaluated the effect of clofibrate treatment on COX-2 inflammatory pathway enzymes and their receptors in breast cancer cells (Figure 5A). Compared to untreated SUM149PT, 24 h treatment with 20 μM clofibrate slightly increased levels of COX-2 (1.1-fold), m-PGES-1 (1.1-fold), and EP1 (1.2-fold) while there was no change in protein levels of EP2 or EP3 and a slightly decreased protein level of EP4 (0.9-fold). However, compared to untreated SUM149PT, a 48 h treatment with 20 μM clofibrate significantly decreased COX-2 inflammatory pathway enzymes and their receptors (Figure 5A) such as COX-2 (0.7-fold), m-PGES-1 (0.4-fold), EP1 (0.8-fold), EP2 (0.7-fold) and EP4 (0.8 fold). There was no significant change in the expression of EP3 (Figure 5A). These results were further confirmed via fluorescent microscopy of COX-2 staining in untreated and 20 μM clofibrate treated SUM149PT and SUM1315 cells (Figure 5B), which showed a definitive decrease in COX-2 expression in the clofibrate treated cells. Similar results were obtained in SUM1315M02 cells as indicated (Figure 5A). These results suggest that clofibrate treatment downregulates COX-2 pathway components in breast cancer cells.

### Clofibrate treatment reduces the 5LO inflammatory pathway enzymes in SUM149PT and SUM1315 cells

**Figure 6:**
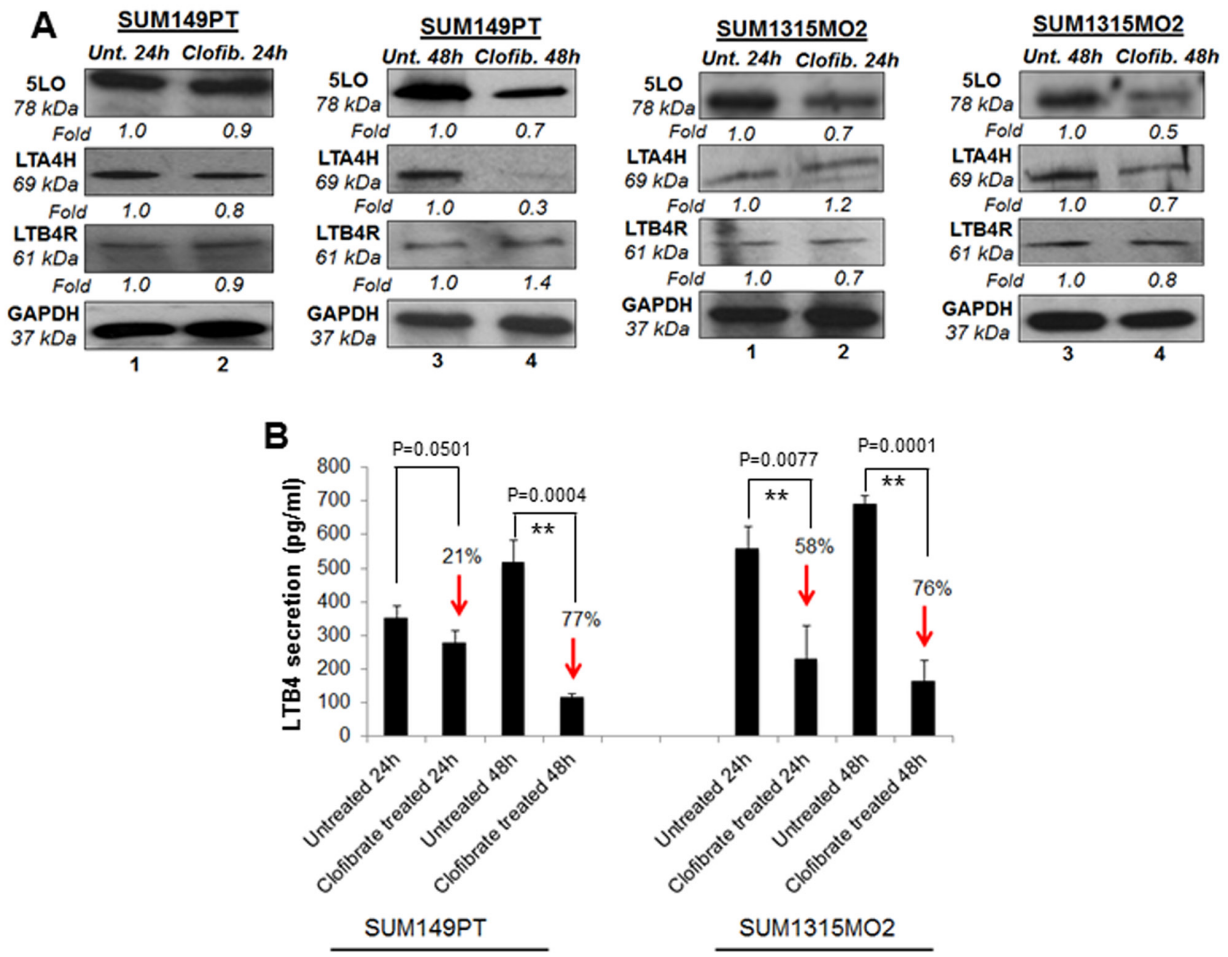
Effect of clofibrate treatment on the 5-lipooxygenase pathway in SUM149PT and SUM1315MO2 cells **A.** SUM149PT and SUM1315MO2 cells were untreated or treated with 20 μM clofibrate and cell lysates were prepared. These cell lysates were Western blotted for 5LO, LTA4H, and LTB4R, and then stripped and re-probed with anti-GAPDH antibody to confirm equal loading. **B.** Effect of clofibrate treatment on LTB4 secretion in SUM149PT and SUM1315MO2 cells. SUM149PT and SUM1315MO2 cells were untreated or clofibrate treated for 24 h and 48 h, and supernatants were collected for LTB4 quantification. Percent inhibition in LTB4 secretion was calculated by considering the secretion from untreated breast cancer cells as 100%. Each bar represents the average +/− SD from three independent experiments. ** represents statistically highly significant.

Since we observed generally lower levels of COX-2 related enzymes with clofibrate treatment in breast cancer cells, we further evaluated the effect of clofibrate treatment on the enzyme and receptor levels of the 5LO inflammatory pathway (Figure 6A and 6B). 20 μM clofibrate treatment significantly reduced 5LO gene expression in SUM149PT and SUM1315MO2 cells (data not shown). While protein levels of the 5LO pathway enzymes such as 5LO and leukotriene A4 hydrolase LTA4H decreased upon clofibrate treatment (Figure 6A). We did not observe any significant change in the leukotriene B4 receptor LTB4R protein level upon clofibrate treatment (Figure 6A). Collectively, our results demonstrate that clofibrate treatment reduces 5-LO inflammatory pathway components.

### Clofibrate treatment inhibits PGE2 and LTB4 secretion from SUM149PT and SUM1315 cells

When cells are activated or exogenous free arachidonate is supplied, PGE2 is synthesized de novo and released into the extracellular space. *In vivo*, PGE2 is rapidly converted to an inactive metabolite (13, 14-dihydro-15-keto PGE2) by the prostaglandin 15-dehydrogenase pathway. COX-2 expression and PGE2 secretion has been shown to accelerate cancer progression via promoting cell adhesion, migration and cell spreading [17]. To evaluate the consequences of overall COX-2 inhibition upon clofibrate treatment, we quantitated PGE2 release (Figure 5C). Clofibrate treatment significantly decreased PGE2 secretion in SUM149PT and SUM1315MO2 (Figure 5C). This decrease was more pronounced in SUM149PT when compared to SUM1315MO2 cells (Figure 5C).

5LO enzyme activation leads to the synthesis and secretion of the chemotactic bioactive lipid metabolite LTB4 [18, 19]. To evaluate the consequences of overall 5-LO inhibition, we quantitated the release of LTB4 upon clofibrate treatment (Figure 6B). 24 h and 48 h treatment of SUM149PT and SUM1315MO2 cells showed a decrease in LTB4 secretion as indicated in Figure 6B. Taken together, results from Figures 5 and 6 indicate that clofibrate treatment downregulates COX-2 and 5LO inflammatory pathways of in breast cancer cells.

### Breast cancer cells express higher levels of fatty acid synthase (FASN) and acetyl coA carboxylase (ACC) as compared to HMEC cells

FASN is the sole mammalian multifunctional enzyme capable of de novo fatty acid synthesis utilizing malonyl-CoA for the first committed step in fatty acid biosynthesis. FASN is increased in obesity and adiposity in humans [20]. FASN overexpression has been associated with a poor prognosis in breast and prostate cancer patients and is an attractive potential target for obesity and cancer therapies [21]. Elevated FASN levels have been identified in breast, prostate, colon, and ovarian cancer patients blood in comparison with normal subjects using ELISA [21]. Another key lipogenic enzyme correlated to cancer etiology and progression is acetyl-coenzyme A carboxylase-1 (ACC1) [22]. It is the rate-limiting enzyme in endogenous fatty acid metabolism catalyzing the condensation of the FASN substrate malonyl-coenzyme A using acetyl-coenzyme A and CO2 as precursors [22]. Next, we tested the level of FASN and ACC1, and the effect of clofibrate treatment on FASN and ACC1 in breast cancer cells.

**Figure 7:**
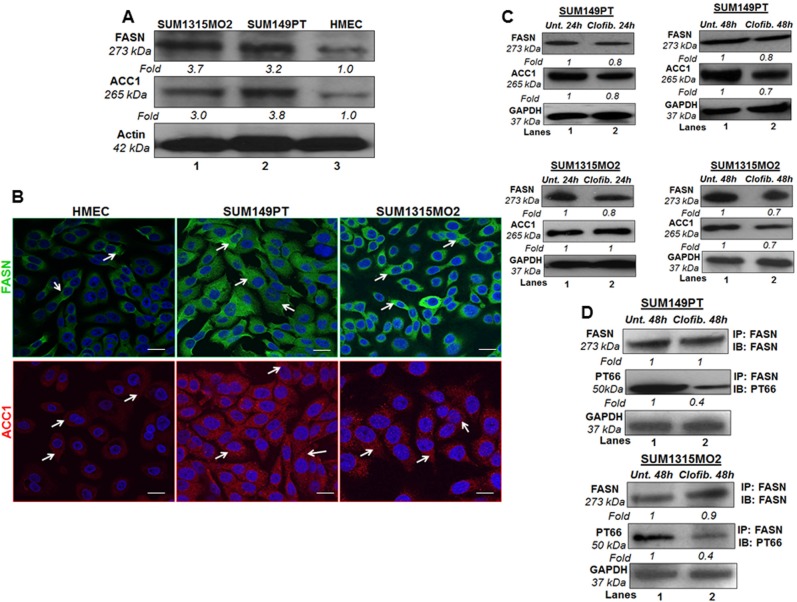
Effect of clofibrate treatment on lipogenic enzymes **A.** FASN and ACC1 protein levels in breast cancer cells and HMEC cells. Lysates prepared from SUM1315MO2, SUM149PT, and HMEC cells were tested for the protein levels of FASN and ACC1. The blots were re-probed with anti-Actin antibody to confirm equal loading. **B.** Immunofluorescence analysis of FASN and ACC1 in SUM149PT, SUM1315MO2, and HMEC cells. Cells were grown to 80%-90% confluence, fixed, permeabilized, and stained with FASN-specific (green) and ACC1-specific (red) antibody. Nuclei were counterstained with DAPI (blue). Magnifications 40X. Scale bar = 20 μm. **C.** Cell lysates prepared from SUM149PT and SUM1315MO2 untreated or treated with 20 μM clofibrate for 24 h and 48 h were Western blotted for FASN and ACC1 and then stripped and re-probed with anti-GAPDH antibody to confirm equal loading. **D.** FASN active/phosphorylated protein levels were measured in untreated or clofibrate treated cell lysates. These lysates were immunoprecipitated with anti-FASN antibody and Immunoblotted with either anti-FASN or anti-PT66 antibody. Equal loading was confirmed by anti-GAPDH antibody. Each blot is a representative of three independent experiments.

Compared to HMEC, breast cancer cells showed increased FASN and ACC1 protein levels in SUM149PT and SUM1315MO2 cells (Figure 7A). Since we observed higher protein levels of FASN and ACC1 in breast cancer cells, we stained SUM149PT, SUM1315MO2 and HMEC cells for FASN and ACC1, and analyzed by confocal microscopy (Figure 7B). SUM149PT and SUM1315MO2 cells showed dense/abundant staining for FASN and ACC1 as compared to HMEC cells (Figure 7B).

### Clofibrate treatment moderately reduced lipogenesis pathway enzymes FASN and ACC but overall reduces the amount of active FASN

After establishing larger amounts of FASN and ACC1 in breast cancer cells, we decided to test the protein levels of these lipogenic enzymes in the presence or absence of clofibrate treatment. Compared to untreated SUM149PT and SUM1315MO2 cells, treatment with 20 μM clofibrate for 24 h and 48 h slightly decreased the protein levels of FASN and ACC1 as indicated (Figure 7C). We next evaluated the effect of clofibrate treatment on the FASN posttranslational modification/FASN tyrosine phosphorylation levels, which are indicators of biologically active FASN fraction. FASN was immunoprecipitated and immunoblotted using a monoclonal phosphotyrosine (PT66) and anti-FASN antibody. Our results indicated that a 48 h treatment with 20 μM clofibrate drastically reduced (60%) the levels of PT66 (0.4-fold) in both SUM149PT and SUM1315MO2 cells (Figure 7D). This suggests that while the overall changes in levels of lipogenic enzymes were not significant, clofibrate treatment markedly reduced the levels of the biologically active form of FASN. However, it remains unclear whether/how FASN is regulated at the post-transcriptional level in SUM149PT and SUM1315MO2 cells.

In many types of cancer, FASN overexpression robustly induces de novo lipogenesis, and the generated lipids are integrated into membrane lipid rafts and activate membrane receptor tyrosine kinases such as the EGFR family, which in turn results in the initiation of oncogenic signaling pathways involving cell survival, proliferation, migration, invasion, and thereby contribute to tumorigenic transformation [23].

### Clofibrate treatment affects various lipid metabolism pathways

**Figure 8:**
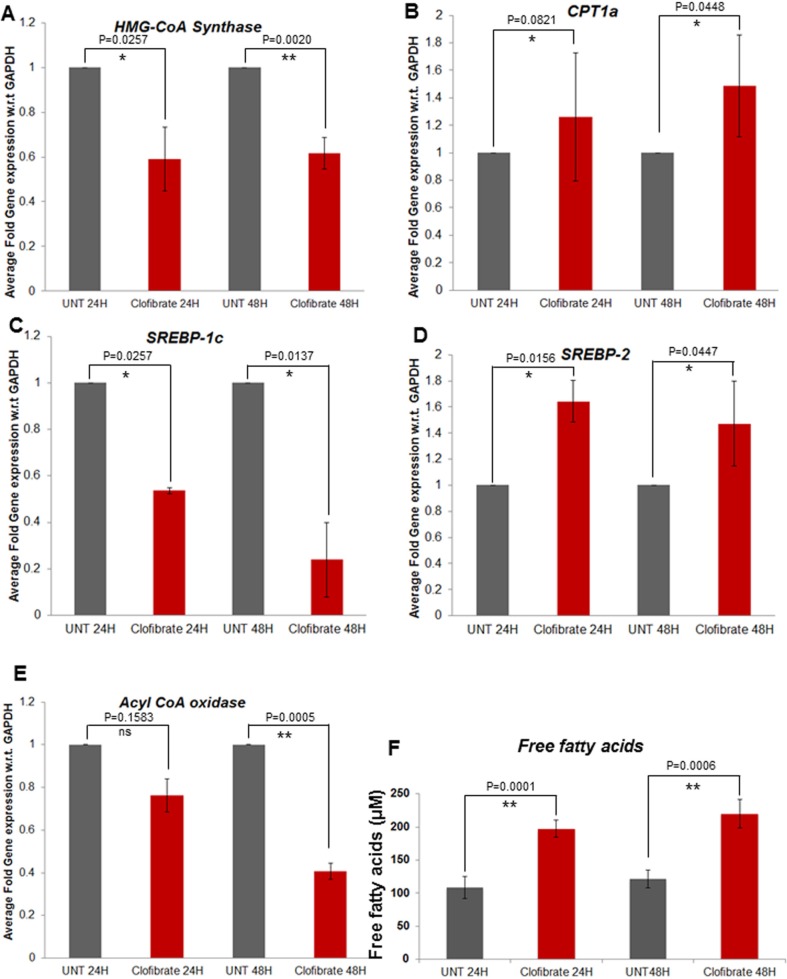
Effect of clofibrate treatment on breast cancer cell lipid metabolism associated genes A–E SUM149PT cells were untreated or treated with 20 μM clofibrate as indicated and RNA was prepared. SREBP-1c, HMG-CoA synthase, Acyl-CoA oxidase, CPT1a and SREBP-2 gene expression was quantitated by real-time RT PCR using their specific primers. Each point represents the average +/− SD from three independent experiments. * denotes statistically significant and ** represents statistically highly significant and ‘ns’ is for non-significant. **F.** Free fatty acid secretion in the supernatants of breast cancer cells untreated or treated with clofibrate for various time points as indicated. ** represents statistically highly significant.

Breast cancer cells expressed higher levels of lipogenic enzymes when compared to normal mammary epithelial cells. Therefore, we focused on a few enzymes involved in lipogenesis (SPTLC1, SCD; stearoyl-CoA desaturase, SREBP-1c, HMG-CoA synthase, Acyl-CoA oxidase) and fatty acid oxidation (CPT-1a and SREBP-2). Compared to untreated, treatment with 20 μM clofibrate significantly decreased the expression of lipogenic enzymes such as SPTLC1 and SPTLC2 (data not shown), Acyl-CoA oxidase, SREBP-1c, and HMG-CoA synthase in SUM149PT cells as indicated (Figure 8A, 8C, 8E). Interestingly, we observed an induction in the gene expression of CPT-1a and SREBP-2, the enzymes involved in fatty acid oxidation in SUM149PT cells (Figure 8B and 8D). Clofibrate treatment significantly induced the secretion of free fatty acid in the supernatants of untreated or clofibrate treated SUM149PT cells (Figure 8F). Similar results were obtained in SUM1315MO2 cells (data not shown).

### PPARα ligand clofibrate treatment inhibits the growth of breast cancer cells

**Figure 9:**
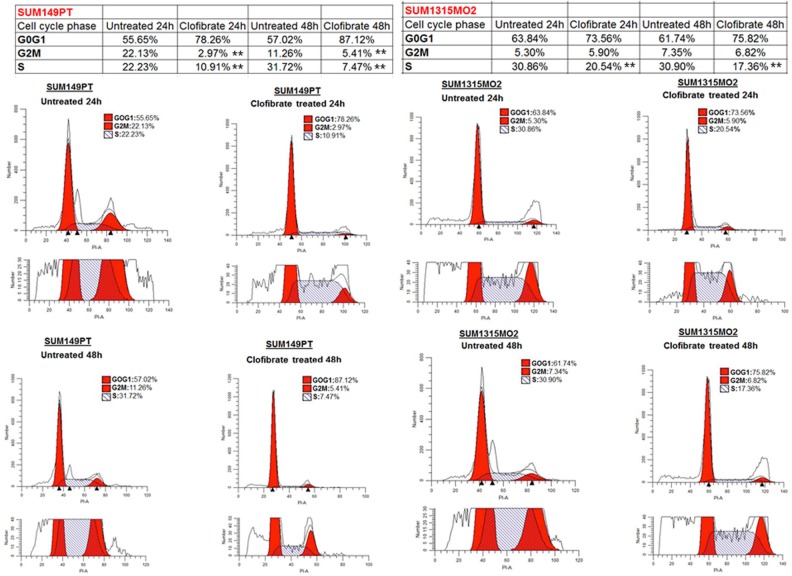
Effect of clofibrate on breast cancer cell cycle events SUM149PT and SUM1315MO2 cells were untreated or treated with 20 μM clofibrate. Cells were collected at 24 h and 48 h post-treatment to examine the cell cycle profile by propidium iodide (PI) staining. The cells were neither replenished with fresh media nor supplemented with the drugs during the 48 h time period. In each representative panel, the horizontal and vertical axis corresponds to the relative DNA content and the number of cells, respectively. The percent of cells in G0G1, S, and G2M phase for untreated and clofibrate treated at the indicated time points was calculated by Modfit 3.2 software. The data is representative of three independent experiments. ** represents statistically highly significant.

To determine whether growth inhibition by clofibrate treatment was attributable to cell cycle arrest, SUM149PT and SUM1315MO2 were untreated or treated with 20 μM clofibrate for 24 h and 48 h (Figure 9). Based on the DNA profile, a higher proportion of untreated SUM149PT and SUM1315MO2 cells were in S-phase compared to clofibrate treated (Figure 9). We observed a distinct anti-proliferative shift in the profile of the cell cycle parameters towards a reduced percentage of cells in the S and G2/M phases, together with a significantly increased percentage of cells in the G0/G1 phase (Figure 9). In SUM149PT cells, it was shown to have approximately an 11% and 24% reduction in S phase at 24 h and 48 h respectively (Figure 9). There was also a roughly 19% and 6% reduction in G2M phase cells at 24 h and 48 h respectively (Figure 9). In SUM1315MO2 cells, we observed approximately a 10% and 13% reduction in S phase at the 24 h and 48 h respectively (Figure 9). However, in SUM1315MO2 cells, there was not much change in the G2/M phase (Figure 9). Overall across both cell types, there was a subsequent cell accumulation in the G0/G1 phase suggesting that clofibrate treatment significantly inhibits breast cancer cells from crossing the G1/S boundary. To confirm the results seen in the cell cycle, we evaluated the level of cell cycle regulatory enzymes and survival kinases.

**Figure 10:**
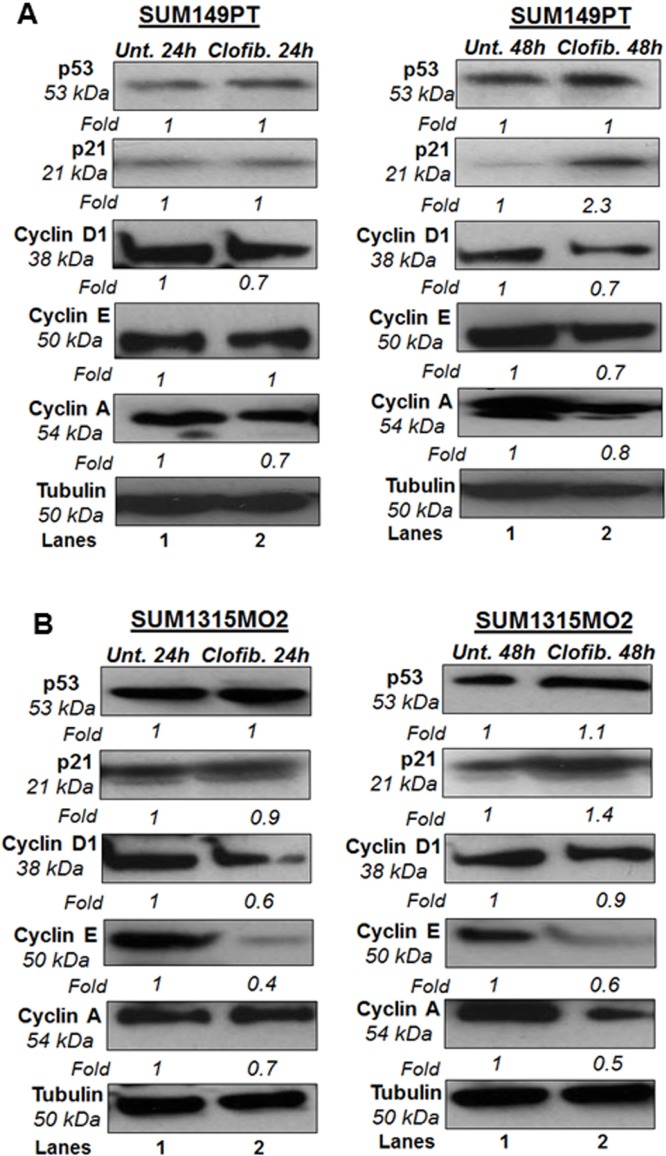
Effect of clofibrate on cell cycle regulatory enzymes Cell lysates prepared from **A.** SUM149PT and **B.** SUM1315MO2 cells that were untreated or treated with 20 μM clofibrate for 24 h and 48 h and Western blotted for p53, p21, cyclin D1, cyclin E, and cyclin A. The blots were re-probed with anti-Tubulin antibody to confirm equal loading. A representative blot from three independent experiments is shown.

SUM149PT and SUM1315MO2 were untreated or treated with 20 μM clofibrate for 24 h and 48 h. Compared to untreated SUM149PT, a 24 h treatment with 20 μM clofibrate induced no significant changes in protein levels of p53, p21, and cyclin E. Other cyclin kinases such as cyclin D1 and cyclin A had decreased protein levels upon clofibrate treatment (Figure 10). Compared to untreated SUM149PT, a 48 h treatment with 20 μM clofibrate resulted in a significant increase of p21 accompanied by reduction in cyclin D1, cyclin E, and cyclin A with no significant change in p53 level (Figure 10). Compared to untreated SUM149PT, a 24 h treatment with 20 μM clofibrate resulted in no significant changes in protein levels of p53 but decreased levels of p21, cyclin D1, cyclin E, and cyclin A (Figure 10). 48 h treatment of SUM149PT cells with 20 μM clofibrate induced p53 and p21 with a subsequent decrease in cyclin D1, cyclin E, and cyclin A levels (Figure 10). The effects were seen more prominently after the 48 h treatment and were apparent in SUM1315MO2 cells (Figure 10). Overall, results indicate that clofibrate treatment exhibits an anti-proliferative effect on breast cancer cells via regulating the level of tumor suppressors, cell cycle inhibitors, and checkpoint kinases.

### Clofibrate treatment inhibits survival kinases in breast cancer cells

**Figure 11:**
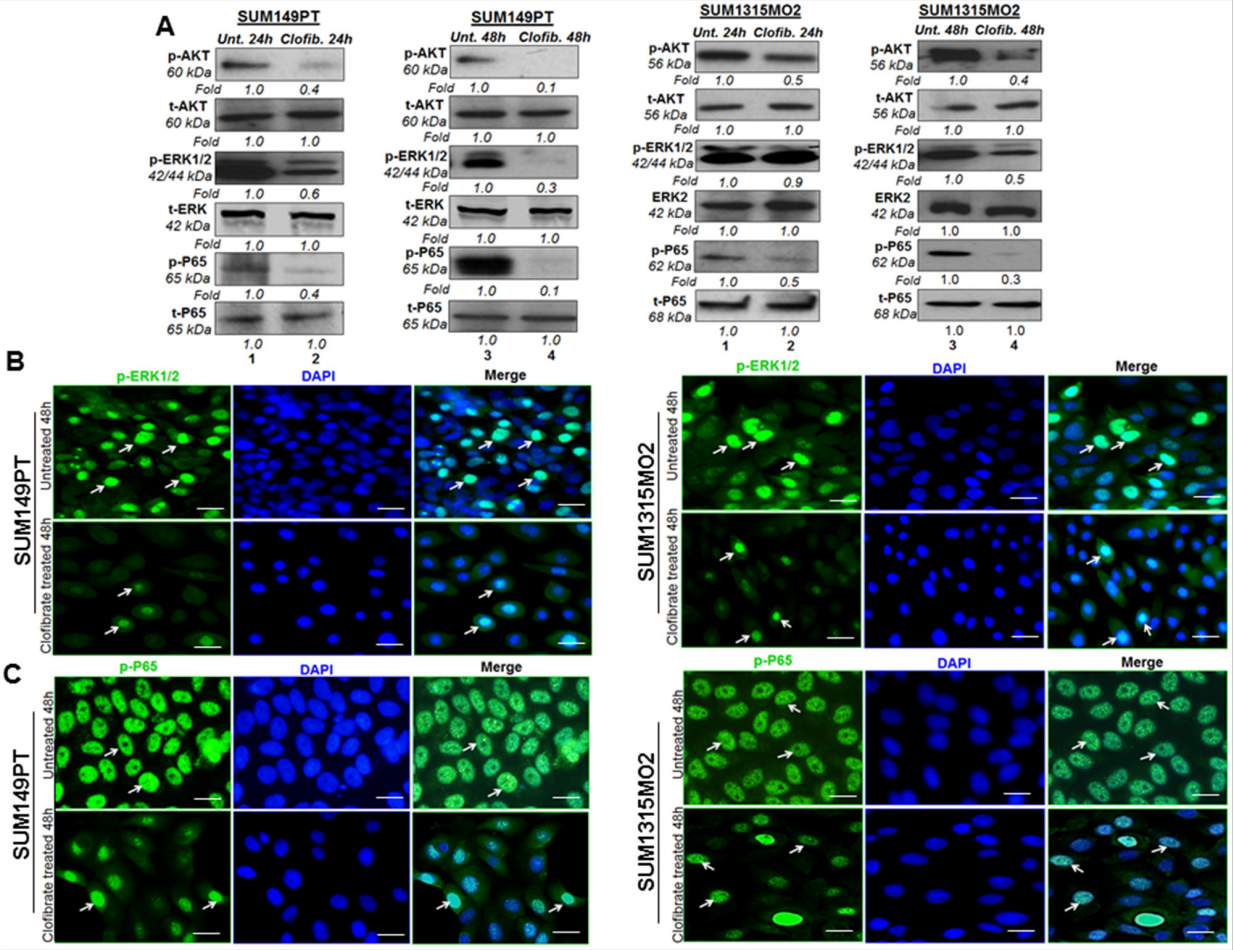
Effect of Clofibrate treatment on cell survival kinases **A.** Whole cell lysates were prepared from SUM149PT and SUM1315MO2 cells that were untreated or treated with 20 μM clofibrate for 24 h and 48 h and Western blotted for P-AKT, P-ERK1/2(p44/42), and P-p65 and normalized with respect to total protein levels. Tubulin was used as the loading control. A representative blot from three independent experiments is shown. **B.** Effect of clofibrate treatment on the nuclear translocation of phosphorylated ERK1/2. Serum-starved SUM149PT and SUM1315MO2 were untreated or treated with 20 μM clofibrate for 48 h in eight-well chamber slides and then collected, permeabilized, and stained with an anti-phospho-ERK1/2 monoclonal antibody. Magnification, 40X. DAPI was used as a nuclear stain and merged with p-ERK1/2 staining. Scale bar = 20 μm. **C.** Clofibrate treatment effect on nuclear translocation of phospho-p65. Serum-starved SUM149PT and SUM1315MO2 cells were untreated or treated with 20 μM clofibrate for 48 h in eight-well chamber slides and then collected, permeabilized, and stained with an anti-p65 polyclonal antibody. Magnification, 40X. DAPI was used as a nuclear stain and merged with p65 staining. Scale bar = 20 μm.

Next, we evaluated the effect of clofibrate treatment on survival kinases such as protein kinase B AKT, extracellular signal related Kinase (ERK), and nuclear factor kappa light chain-enhancer of activated B cells (Nf-kB). SUM149PT and SUM1315MO2 (Figure 11A) were untreated or treated with 20 μM clofibrate for 24 h and 48 h. Compared to untreated cells, a 24 h treatment with 20 μM clofibrate significantly reduced protein levels of p-AKT, p-ERK1/2, and p-P65. When examining the untreated SUM149PT, a 48 h treatment with 20 μM clofibrate showed a drastic decrease in the survival kinase pathway enzymes as seen in p-AKT, p-ERK1/2, and p-P65 (Figure 11A). Similarly, compared to untreated SUM149PT, a 24 h treatment with 20 μM clofibrate decreased protein levels of p-AKT, p-ERK1/2, and p-P65. When examining the untreated SUM149PT, a 48 h treatment with 20 μM clofibrate significantly decreased p-AKT, p-ERK1/2, and p-P65 (Figure 11A). All fold calculations of phosphorylated enzyme survival kinase forms were normalized against the total levels of AKT, ERK and p65. Similarly, we observed downregulation of various signaling pathways upon clofibrate treatment of SUM1315MO2 cells as indicated (Figure 11A).

### Clofibrate treatment inhibits rapid nuclear translocation of p-65 and p-ERK1/2

Phosphorylation and dephosphorylation play significant roles in the signaling cascades, and the subcellular location of a phosphorylated protein is important for its activity and inactivity. The p44 and p42 MAPK isoforms (ERK1 and ERK2) are serine-threonine kinases, and their activity is stimulated by phosphorylation mediated by MEK1 and MEK2, which activate their kinase activity. ERK1 and ERK2 have been shown to be the only key mediators of signal transduction transmitting signals from the cell surface to the nucleus. Upon signal induction, MEK remains cytoplasmic, whereas ERKs anchored to MEK in the cytoplasm of resting cells translocate to the nucleus, a process which is rapid, reversible, and controlled by the strict activation of the MAPK cascade [24, 25]. Clofibrate treatment induced inhibition of ERK1/2 activation as examined by using monoclonal antibody against the MAP kinase synthetic diphosphopeptide. This antibody specifically recognized the active, doubly phosphorylated forms but not the inactive mono- and nonphosphorylated forms of ERKs [26]. Treatment of SUM149PT with 20 μM clofibrate for 48 h inhibited the rapid nuclear translocation of phosphorylated ERK1/2 with only 18% translocated compared to 40% translocation in untreated cells (Figure 11B). Treatment of SUM1315MO2 with 20 μM clofibrate for 48 h inhibited the rapid nuclear translocation of phosphorylated ERK1/2 with only having 10% translocated compared to 36% translocation in untreated cells (Figure 11B). As stated, a larger amount of phosphorylated p24/p44 MAPKs were detected in the nuclei in untreated cells than in treated cells. These results demonstrate that clofibrate treatment inhibits rapid nuclear entry of phosphorylated p42/p44 MAPKs in breast cancer cells.

NF-κB belongs to a highly conserved family of transcription factors with an N-terminal Rel homology domain and a C-terminal transactivation domain that includes c-Rel, p50 (NF-κB1), p52 (NF-κB2), p65 (RelA), and RelB [27]. Each of these polypeptides can form homodimers or dimerize with other Rel family members, and the prototype NF-κB is composed of p50 and p65. Once activated in a stimulus-specific manner, NF-κB rapidly translocates into the nucleus and induces the transcription of various cellular genes [27]. Treatment of SUM149PT with 20 μM clofibrate for 48 h inhibited the rapid nuclear translocation of p65 with only having 50% translocated compared to 100% translocation in untreated cells (Figure 11C). Treatment of SUM1315MO2 with 20 μM of clofibrate for 48 h inhibited the rapid nuclear translocation of NF-κB-p65 with only 29% translocation compared to 100% translocation in untreated cells (Figure 11C). As stated, a larger amount of phosphorylated p65 was detected in the nuclei in untreated cells than in treated cells. These results demonstrate that clofibrate treatment inhibits rapid nuclear entry of NF-κB-p65 in breast cancer cells.

### Clofibrate treatment induced the level of coactivator proteins in the nuclear complexes of SUM149PT and SUM1315MO2 cells

**Figure 12:**
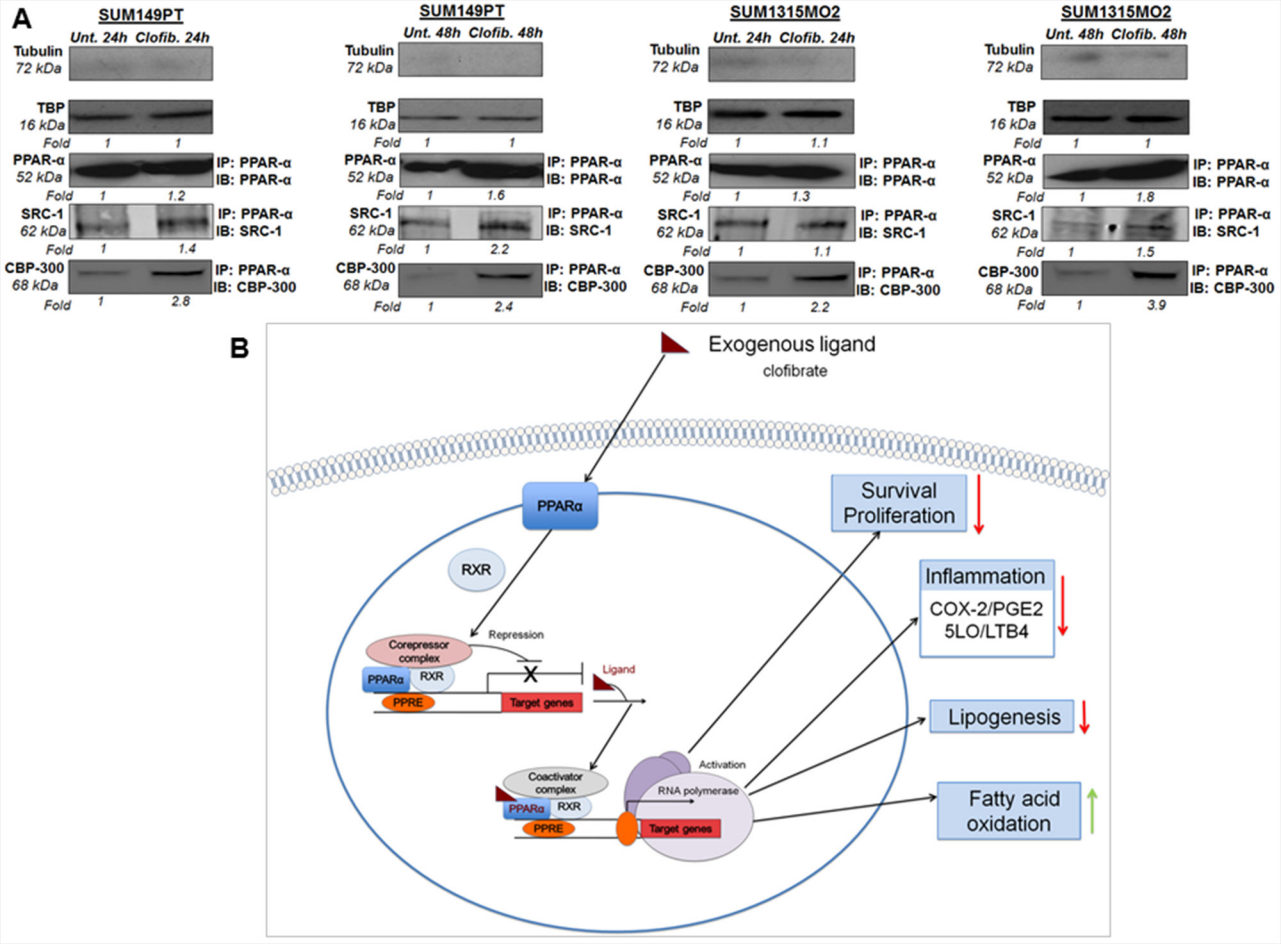
A proposed model for the role of PPARα agonist clofibrate in the regulation of inflammatory and lipid pathways in breast cancer cells **A.** Effect of clofibrate treatment on coactivator proteins in the nuclear complexes of SUM149PT and SUM1315MO2 cells. Nuclear complexes were prepared from SUM149PT and SUM1315MO2 that were untreated or treated with 20 μM clofibrate for 24 h and 48 h and immunoprecipitated with PPARα antibody, and Western blotted for SRC-1 and p300/CBP, and normalized with respect to the levels of total protein levels. Tubulin was used as the loading control. **B.** The present study indicates that highly metastatic form of breast cancer cell lines including SUM149PT and SUM1315MO2 cells express tremendous level of PPARα along with abundant expression and activity of inflammatory pathways of COX-2 and 5-LO. We demonstrated that SUM149PT and SUM1315MO2 cells are metabolically active, express the active form of FASN, and secrete free fatty acids in their tumor microenvironment and FASN/lipogenic pathways rich phenotype. Our study unraveled that PPARα activation in breast cancer cells downregulated COX-2 and 5-LO inflammatory pathways. Clofibrate treatment reduced lipogenic enzymes (FASN) and induced enzymes involved in fatty acid oxidation (CPT-1a and SREBP-2). PPARα activation via clofibrate showed anti-proliferative effects in breast cancer cells via inhibition of survival kinases (NF-kB and ERK1/2), cell cyclin kinases, and induction of p21. Clofibrate treatment modulated the expression of PPRE containing target genes via induction of coactivators (SRC-1 and p300/CBP) in nuclear complexes probably binding to PPARα. Red arrow represents inhibition and green arrow represents induction in the schematic.

Ligand treatment is known to mediate activation of PPARs via dissociation of corepressors and concomitant association with coactivators, such as SRC1 and CBP/p300 [11]. In order to test whether clofibrate treatment activates PPARα via association with coactivators, we prepared nuclear complexes from untreated or 20 μM clofibrate treated SUM149PT and SUM1315MO2 for 24 h and 48 h. Nuclear complexes were tested for their purity by absence of tubulin and presence of the TATA binding protein (TBP) (Figure 12A). Compared to untreated cells, 20 μM clofibrate treated SUM149PT and SUM1315MO2 for 24 h and 48 h nuclear complexes immunoprecipitated with PPARα antibody showed higher expression of SRC1 and CBP/p300 (Figure 12A).

## DISCUSSION

Although there has been significant interest in understanding the role of PPARα in metabolic disorders, there are only a few reports on PPARα in human malignant diseases especially inflammatory and invasive breast cancer. In this study, we made several interesting findings in the context of PPARα nuclear receptor signaling, lipogenic, and inflammatory pathways in inflammatory and invasive breast cancer cells. Here, we tested the effect of three fibrate drugs including clofibrate, fenofibrate, and WY14643 on inflammatory and invasive breast cancer cells. We used triple negative breast cancer (TNBC) cell lines, which lines lack three characteristic molecular markers including estrogen receptor (ER), progesterone receptor (PR), and do not have amplification of HER-2/Neu [28]. TNBC represents approximately 10–15% of all breast cancers and patients with TNBC have a poor outcome compared to the other subtypes of breast cancer since they lack validated molecular targets [28].

Based on the results obtained from the cytotoxicity and MTT assays performed on the control HMEC cell line and breast cancer cells lines including SUM149PT and SUM1315MO2, we chose to study the role of clofibrate. Clofibrate was the first fat and triglyceride lowering fibrate, developed in Japan in the 1960s. We demonstrate that clofibrate suppressed the growth of breast cancer cells in conjunction with the reduction of inflammatory (COX-2/5LO), lipogenic pathways, and a significant induction of genes involved in fatty acid oxidation. A direct correlation exists between elevated COX-2/PGE2, 5LO/LTB4, and the pathogenesis of colorectal, prostate, lung and breast cancers, as well as several hematological malignancies including chronic lymphocytic leukemia, Hodgkin's and non-Hodgkin's lymphomas, and multiple myeloma [29–32].

Activation of PPARα in breast cancer cells suppressed inflammatory COX-2 and 5LO activity and resulted in decreased PGE2 and LTB4 secretion as well as a reduction in PGE2 and LTB4 receptor expression. Interestingly, we observed PGE2 levels significantly increase between 24–48 h quite prominently in the SUM149PT cell line, however, not so much in the SUM1315MO2 cell line. This probably could be due to a slow burst of PGE2 secretion accompanied by signal transduction activation, especially NF-KB, which can then drive the COX-2 promoter activation [33] leading to second storm of PGE2 in the SUM149PT cells. Clofibrate treatment of breast cancer cells effectively inhibited cell survival and cell cycle-related kinases.

FASN is minimally expressed in most normal human tissues because it appears to use preferentially circulating fatty acids for the synthesis of new structural lipids [22]. Interestingly, a biologically aggressive subset of carcinomas constitutively express high levels of FASN and undergo significant endogenous fatty acid biosynthesis independently of the regulatory signals that downregulate fatty acid synthesis in normal cells, and upregulation of FASN gene expression is an early event in cancer development that is more pronounced in advanced tumors [22]. FASN is a potential integrative metabolic mediator regulated by glucose, insulin, amino acids, fatty acids, leptin, and other metabolites, which serve as endogenous activators of the nuclear receptor PPARα [20]. ACC1, another enzyme of the lipogenic pathway, is known to induce a marked increase of endogenous lipogenesis in prostate and breast cancer cells [22]. We demonstrated that breast cancer cells express abundant levels of FASN and ACC1. Even though no functional PPRE has been identified in the promoter region of the FASN gene [34, 35], many studies have reported modulation of FASN gene activity or expression in liver and adipose tissue, and are tissue specific [36]. Therefore, we analyzed whether clofibrate treatment stimulates/suppresses FASN in breast cancer cells. We observed reduction in the biologically active/phosphorylated fraction of FASN upon clofibrate treatment.

In response to fibrates, PPAR*α* heterodimerizes with retinoid X receptor-*α* (RXR-*α*), and stimulate the transcription of genes containing PPREs in their promoter sequence [11]. Since fibrates metabolize fatty acids and triglycerides by stimulating peroxisomal *β*-oxidation [12, 13], we tested a few PPRE containing fatty acid oxidation pathway genes. Clofibrate treatment efficiently controlled the expression of various PPRE harboring lipogenic and fatty acid oxidation pathway genes such as SREBP-1c, SREBP-2, HMG-CoA synthase 2, Acyl-CoA oxidase, and CPT-1a. FASN in prostate cancer cells also seems to be mediated by the SREBP pathway [37]. Activation of the key lipogenic transcription factor SREBP-1c enhances the expression of one of its primary lipogenic target enzymes FASN by stimulating the transcriptional activity of the FASN promoter that harbors a complex SREBP-binding site [38]. Reduction of SREBP-1c upon clofibrate treatment in breast cancer cells further adds to the anti-lipogenic potential of PPARα nuclear receptor signaling pathway. Our results show that clofibrate treatment not only downregulates the genes involved in lipogenesis, but it also induces CPT-1a, a gene of fatty acid oxidation. CPT-1a is the first and rate-limiting step of fatty acid transport into mitochondria for oxidation to carbon dioxide.

The fact that PPARα agonists are reported to inhibit tumor growth in various cancer model systems [2, 3] led us to examine how activation of PPARα might affect the growth of breast cancer cells. We have shown that the PPARα agonist clofibrate diminishes the level and activation of key survival kinases such as Nf-KB and ERK1/2 in breast cancer cell lines. We demonstrated that PPARα activation decreased the growth rate of breast cancer cells via reducing the level of various cell-cycle regulating cyclins. This is the first demonstration that activation of PPARα ligand suppresses expression and activity of survival kinases in breast cancer cells, thus providing novel insight into the nuclear receptor mediated signaling pathways involving highly metastatic breast cancer.

Although the possibility has been reported that the PPARα ligands could reduce growth of some types of malignant tumors and prevent carcinogenesis [2, 3, 12, 13], the mechanism remains unresolved. Interestingly, we found that expression of coactivators increased in the nuclear complexes, suggesting that PPARα nuclear receptor signaling is active upon clofibrate treatment. It is unclear at this point whether there are changes or decreases in the level of various transcription corepressors such as nuclear receptor corepressor (NCoR) and silencing mediator for retinoid and thyroid hormone receptor (SMRT), which are known to inhibit nuclear receptor signaling [11]. Our current findings provide a mechanistic explanation of how PPARα agonists could act as effective anti-inflammatory and anti-proliferative agents for breast cancer cells.

The prolonged use of some fibrates has been reported to cause peroxisome proliferation subsequently leading to hepatomegaly and tumor formation in the liver of rodents [39]. Since induction of hepatic tumor promotion by fibrate drugs has not been demonstrated in humans [40], other primates or guinea pigs, fibrates at a low dose taken for shorter duration of time or used as combination therapy could have anti-tumorigenic effects [11, 41]. Humans have considerably lower levels of PPAR*α* in the liver than in rodents, which, in part, explains the species differences in the carcinogenic response to peroxisome proliferators, and suggests hepatic tumor formation not be a concern in humans.

In conclusion, we have demonstrated that activation of PPARα via its agonist clofibrate downregulates the inflammatory and lipogenic pathways along with suppressing the growth of human breast cancer cells. These findings provide new insights into our understanding of the nuclear receptor signaling pathways in inflammatory breast cancer cells and support the use of PPARα agonists as therapeutic anticancer agents. Our study would set the basis for future studies designed to validate in *in vivo* efficacy of PPAR*α* ligands as anti-tumorigenic agents in breast cancer models. Though, PPARα, a ligand-activated nuclear receptor/transcription factor, is a key negative regulator of inflammation whereas PPARα deficient mice exhibit enhanced inflammation and rodent tumorigenesis [42]. Profiles of PPARα ^−/−^ mice have been reported to reveal defects in energy regulation, fatty acid catabolism and carnitine homeostasis [43, 44]. Currently, the PPAR-α genetic variants and knock out studies in cancer biology are few; however, the expanding use of next-generation DNA sequencing technologies, including chromatin immunoprecipitation followed by DNA sequencing and global DNA methylation analysis will allow the identification of epigenetic modifications that may contribute to tumor progression and oncogenesis [44].

## MATERIALS AND METHODS

### Cell culture

Primary human mammary epithelial cells (HMEC) (830-05a, Cell Applications, San Diego, CA) were cultured in HMEC medium (815-500, Cell Applications). Primary inflammatory breast cancer cells, SUM149PT (Asterand, Detroit, MI), and highly Invasive Breast Cancer cells, SUM1315MO2 (Asterand) were grown in F-12 media (11765-054, Gibco BRL, Grand Island, NY) supplemented with 10% heat-inactivated fetal bovine serum (HyClone, Logan, UT), insulin (19278, Sigma, St. Louis, MO), HEPES (H3375; Sigma), EGF (E9644; Sigma) for SUM1315MO2 and Hydrocortisone (H4001, Sigma) for SUM149PT. All cells were tested for mycoplasma contamination by the standard Limulus assay (Limulus amebocyte lysate endochrome; Charles River Endosafe, Charleston, SC) method as per manufacturer's instructions. All cells were cultured in LPS-free medium.

### Reagents

Antibody against PPARα was from Abcam. P-p65, P65, AKT, P-AKT, P-p44/42, Erk2, FASN, ACC1, P53, P21, cyclin A, cyclin E, and GAPDH antibodies were from Cell Signaling Technology, Inc., Danvers, MA. Antibodies used against β-actin, FASN tyrosine phosphorylation (PT66), and tubulin, were from Sigma. 5-LO, LTA4H, COX-1 and COX-2 antibodies were from Cayman Chemicals, Ann Arbor, MI.

### Gene expression profiling by quantitative real time-PCR

Total RNA was isolated with TRIzol Reagent (Life Technologies Corporation, Grand Island, NY) and treated with DNase I (Life Technologies Corporation) at 37°C for 30 min. Reverse transcription was performed using a High-Capacity cDNA reverse transcription kit (Life Technologies Corporation) and converted to cDNA, relative abundance of target gene mRNA was measured by qRT-PCR using the deltadelta method (ratio, 2[DCt sample-DCt control]) as described previously [19]. Transcripts of the genes of interest were detected by real-time RT-PCR using gene-specific primers (Table 1) as per procedures described previously [19]. Normalization was done with respect to GAPDH mRNA levels.

**Table 1:** Sequences of real time primers used in the study

Primer	Orientation	Sequence (5′-3′)
**GAPDH**	Sense	GAAGGTGAAGGTCGGAGTC
	Antisense	GAAGATGGTGATGGGATTTC
**18S**	Sense	AACCCGTTGAACCCCATT
	Antisense	CCATCCAATCGGTAGTAGCG
**HPRT**	Sense	GGACAGGACTGAACGTCTTGC
	Antisense	CTTGAGCACACAGAGGGCTACA
**HMG-CoA Synthase**	Sense	GAATCAGTGGAAGCAAGCTGG
	Antisense	GAATCAGTGGAAGCAAGCTGG
**CPT1a**	Sense	TCCAGTTGGCTTATCGTGGTG
	Antisense	CTAACGAGGGGTCGATCTTGG
**SREBP-1c**	Sense	GGAGCCATGGATTGCACATT
	Antisense	GCTTCCAGAGAGGAGGCCAG
**SREBP-2**	Sense	CCCTTGACTTCCTTGCTGCA
	Antisense	GCGTGAGTGTGGGCGAATC
**Acyl CoA Oxidase**	Sense	AGTGCCCAGATGATCTTGAAGC
	Antisense	CTGCCAGAGGTAACCATTTCCT
**SCD-1**	Sense	TGGGTTGGCTGCTTGTG
	Antisense	GCGTGGGCAGGATGAAG
**FASN**	Sense	CTTCCGAGATTCCATCCTACGC
	Antisense	TGGCAGTCAGGCTCACAAACG
**PPAR-α**	Sense	CTATCATTTGCTGTGGAGATCG
	Antisense	AAGATATCGTCCGGGTGGTT

**Table 2:** Abbreviations used throughout the manuscript

**COX-2**	Cyclooxygenase-2
**5-LO**	5-Lipoxygenase
**RXR**	Retinoid X receptor
**PPAR**	Peroxisome proliferator-activated receptor
**FASN**	Fatty acid synthase
**PPRE**	Peroxisome proliferator response element
**IBC**	inflammatory breast cancer
**ACC1**	Acetyl-coenzyme A carboxylase 1
**SCD**	Stearoyl-CoA desaturase
**SREBP-1c**	Sterol regulatory element-binding transcription factor-1c
**SPTLC1**	Serine Palmitoyltransferase, Long Chain Base Subunit 1
**ERK**	Extracellular signal related kinase
**Nf-kB**	Nuclear factor kappa light chain-enhancer of activated B cells
**PGE2**	Prostaglandin E2
**LTB4**	Leukotriene B4
**CPT-1a**	Carnitine Palmitoyltransferase 1a
**NCoR**	Nuclear receptor corepressor
**HMEC**	Primary human mammary epithelial cells
**ACS**	Acyl CoA Synthetase
**ACO**	Acyl CoA Oxidase
**NEFA**	Non-esterified fatty acids
**SRC-1**	Steroid Receptor Coactivator-1 a
**p300/CBP**	p300 kDa/CREB binding protein
**LTA4H**	Leukotriene A4 hydrolase
**LTB4R**	Leukotriene B4 receptor
**EGFR**	Epidermal growth factor receptor
**SMRT**	Silencing mediator for retinoid and thyroid hormone receptor
**SPTLC1**	serine palmitoyttransferase long-chain
**FFA**	Free fatty acids
**AA**	Arachidonic acid
**MLYCD**	Matonyl-CoA decarboxytase
**PI**	Propidium iodide
**IFA**	Immunofluorescence assay
**IHC**	Immunohistochemistry
**LDH**	Lactate dehydrogenase

### Immunofluorescence assay (IFA)

HMEC, SUM149PT, and SUM1315MO2 cells were seeded in eight-well chamber slides (Nalge Nunc International, Naperville, IL). For immunostaining, cells were fixed with 4% paraformaldehyde (PFA), permeabilized with 0.4% Triton-X 100 and stained with primary antibodies overnight at 4°C. Cells were washed and developed with Alexa 594 or Alexa 488-coupled secondary antibody (Molecular Probes, Eugene, OR), and the nuclei were visualized using DAPI (Ex358/Em461; Molecular Probes) as counter stain. Stained cells were washed and viewed with appropriate filters on an Olympus Confocal laser-scanning microscope (Fluoview FV10i) with the metamorph digital imaging system [19].

### Immunohistochemistry (IHC)

Sections from breast tissue samples of healthy subjects and patients were obtained from Biochain Institute, Inc. (breast tumor tissue array Z7020007). The tumor diagnosis and tumor grading (stages I-III) for the breast cancer tissue was done by Biochain Institute Inc. This is a 16 patient breast cancer tissues array of a specific type of breast cancer i.e. the invasive ductal carcinoma. The sample distribution was hugely varied with the tumor staging from T1N0M0 to T4N1M0 (T indicates the primary tumor, N indicates the regional lymph node metastasis and M indicates distant metastasis), the tumor grade varying from grade I-III and the age of the patients ranging from 28 to 77 years. There was no information regarding the ethnicity of the patient samples. Additionally, the two control tissue samples that were age matched and part of the normal tissue were taken out during surgical resection as well. These tissues are used as samples for staining control and comparison. Inflammatory breast cancer tissue sample (breast tumor tissue array T22350862-2) was obtained from Biochain as well. Permission was obtained according to the Declaration of Helsinki and following the specific authorization of the local Institutional Review Board (IRB) Committee of Chicago Medical School, Rosalind Franklin University of Medicine and Science. Since the tissue sections were commercially obtained from the Biochain Institute, Inc., each sample was anonymous and blinded for laboratory research use. IHC was performed using primary antibodies against human PPARα with no cross-reactivity with PPARβ or PPARγ (anti-mouse PPARα from Millipore, Temecula, CA) or FASN (Sigma) using the protocols as described previously [19].

### Cell viability assay

The viability of the cells after treating with fenofibrate, WY14643, and clofibrate were determined by lactate dehydrogenase (LDH) measuring cytotoxicity assay [19, 45]. LDH is a cytosolic enzyme that is an indicator of cellular toxicity. LDH is released into cell culture media when the plasma membrane is damaged. In the first step, LDH catalyzes the reduction of NAD^+^ to NADH and H^+^ by oxidation of lactate to pyruvate. In the second step of the reaction, diaphorase uses the newly formed NADH and H^+^ to catalyze the reduction of a tetrazolium salt to highly colored formazan, which absorbs strongly at 490 nm. The assay measures extracellular LDH in culture media using an enzymatic reaction that results in a red formazan product which can be measured spectrophotometrically.

### Cell cycle analysis by flow cytometry

SUM149PT and SUM1315MO2 cells were untreated or treated with clofibrate for 24 h or 48 h and used for cell cycle analysis [19]. Cells were fixed with 70% methanol overnight and DNA was stained with propidium iodide (PI) at a final concentration of 50 μg/ml with RNaseA (100 U/ml) prior to flow cytometry analysis using an LSRII (BD Biosciences). Results were analyzed using ModFit Lt V3 software (Verity Software House).

### Proliferation assay

The proliferating index of untreated or clofibrate treated cells was determined by the 3-(4, 5-dimethylthiazol-2-yl)-2, 5-diphenyl tetrazolium bromide; MTT) based colorimetric assay (ATCC, Manassas, VA) as described previously [19]. The amount of MTT (yellow tetrazolium salt) that is converted to insoluble purple formazan crystals in the metabolically active cells presents the number of proliferating cells. The MTT Cell Proliferation Assay measures the cell proliferation rate and conversely, when metabolic events lead to apoptosis or necrosis, the reduction in cell viability.

### Western blot analysis

Cell lysates were quantitated by BCA assay and equal amounts of protein (40 μg/lane) were separated by SDS-PAGE, electrotransferred to 0.45-mm nitrocellulose membranes, blocked with 5% BSA, probed with antibodies of interest, and visualized using an enhanced-chemiluminescence (ECL) detection system [19].

### ELISA for LTB4 and PGE2

LTB4 and PGE2 levels in the supernatants of untreated or 20 μM clofibrate treated breast cancer cells were measured by enzyme-linked immunosorbent assay (ELISA; R&D Systems, Minneapolis, MN) as described previously [18, 19, 46]. Data are expressed as the amount of LTB4 or PGE2 produced (pg/ml) per 10^5^ cells.

### DNA-binding activity of PPARα

The PPARα binding activity assay was performed by using a Trans-AM ELISA based kit from Active Motif (Carlsbad, CA, USA) according to the manufacturer's protocol. Briefly, nuclear extracts were incubated in a 96-well plate coated with an oligonucleotide containing the PPRE motif (5′-AACTAGGTCAAAGGTCA-3′). PPARα contained in the nuclear extract, specifically bound to the immobilized oligonucleotide, was detected by using an anti-PPARα antibody followed by a secondary HRP- (horseradish peroxidase-) conjugated antibody in an ELISA based assay [19].

### Free fatty acid assay

Triglycerides (TAG) are the digestive end product of breaking down dietary fats, and serve as an energy source and play a key role in metabolism. Secreted enzyme lipases hydrolyze the triglyceride ester bond, yielding glycerol and free fatty acids (FFA) in a process called lipolysis. Measurement of free fatty acids is important in metabolic diseases and cancer. We quantitated FFAs by using Free Fatty Acid Assay Kit (Cell Biolabs, San Diego, CA) that measures non-esterified fatty acids (NEFA) in serum and plasma by a coupled enzymatic reaction system (ACS-ACO Method). First, Acyl CoA Synthetase (ACS) catalyzes fatty acid acylation of coenzyme A. Next, the acyl-CoA product is oxidized by Acyl CoA Oxidase (ACO); producing hydrogen peroxide, which reacts with the colorimetric probe and gives absorbance at 570 nm. Palmitic acid was used as standard.

### Statistical analysis

Three independent experiments were performed for each experiment to obtain reproducible results. The representative histograms are the average ± SD of three independent experiments. The statistical significance of differences between experimental groups was determined by Student's *t* test. Statistical significance was calculated using GraphPad Prism 5 software.
